# Antecedent infections in Guillain‐Barré syndrome in endemic areas of arbovirus transmission: A multinational case‐control study

**DOI:** 10.1111/jns.12469

**Published:** 2021-09-30

**Authors:** Sonja E. Leonhard, Cheng Yin Tan, Annemiek A. van der Eijk, Ricardo R. Reisin, Suzanne C. Franken, Ruth Huizinga, Samuel Arends, Manou R. Batstra, Selma M. Bezerra Jeronimo, Judith Drenthen, Laura de Koning, Luciana Leon Cejas, Cintia Marchesoni, Wilson Marques, Nortina Shahrizaila, Dardo F. Casas, Andrea Sotelo, Belen Tillard, Mario‐Emilio Dourado, Bart C. Jacobs

**Affiliations:** ^1^ Department of Neurology Erasmus MC, University Medical Center Rotterdam The Netherlands; ^2^ Department of Medicine University of Malaya Medical Center Kuala Lumpur Malaysia; ^3^ Department of Viroscience Erasmus MC, University Medical Center Rotterdam The Netherlands; ^4^ Department of Neurology Hospital Británico Buenos Aires Argentina; ^5^ Department of Immunology Erasmus MC, University Medical Center Rotterdam The Netherlands; ^6^ Department RH‐MDC – Immunology Reinier de Graaf Gasthuis Delft The Netherlands; ^7^ Institute of Tropical Medicine of Rio Grande do Norte Federal University of Rio Grande do Norte Natal Brazil; ^8^ Department of Neurology Hospital das Clinicas da Faculdade de Medicina Ribeirão Preto Brazil; ^9^ Department of Neurology Hospital Dr Enrique Vera Barros La Rioja Argentina; ^10^ Department of Neurology Sanatorio Adventista del Plata Entre Rios Argentina; ^11^ Department of Neurology Sanatorio Los Arcos Buenos Aires Argentina; ^12^ Department of Neurology Hospital Universitário Onofre Lopes Natal Brazil

**Keywords:** anti‐ganglioside antibodies, anti‐glycolipid antibodies, chikungunya virus, dengue virus, Guillain‐Barré syndrome, Zika virus

## Abstract

Half of the world's population is at risk of arthropod‐borne virus (arbovirus) infections. Several arbovirus infections have been associated with Guillain‐Barré syndrome (GBS). We investigated whether arboviruses are driving GBS beyond epidemic phases of transmission and studied the antibody response to glycolipids. The protocol of the International Guillain‐Barré syndrome Outcome Study (IGOS), an observational prospective cohort study, was adapted to a case‐control design. Serum samples were tested for a recent infection with Zika virus (ZIKV), dengue virus (DENV), chikungunya (CHIKV) virus, hepatitis E virus, Epstein‐Barr virus (EBV), cytomegalovirus (CMV), *Campylobacter jejuni*, and *Mycoplasma pneumoniae*, and for antibodies to glycolipids. Forty‐nine patients were included from Brazil (63%), Argentina (14%), and Malaysia (22%). Evidence of a recent infection was found in 27/49 (55%) patients: *C jejuni* (n = 15, 31%), *M pneumoniae* (n = 5, 10%), CHIKV (n = 2, 4%), EBV (n = 1, 2%), *C jejuni* and *M pneumoniae* (n = 2, 4%), CMV and DENV (n = 1, 2%), and *C jejuni* and DENV (n = 1, 2%). In 22 patients, 35 paired controls were collected. Odds ratio for recent infections did not significantly differ between cases and controls. No typical anti‐ganglioside antibody binding was associated with recent arbovirus infection. We conclude that arbovirus infections occur in GBS patients outside of epidemic viral transmission, although not significantly more than in controls. Broad infection and anti‐ganglioside antibody serology are important to establish the most likely pathogenic trigger in GBS patients. Larger studies are necessary to determine the association between arboviruses and GBS.

## 1 INTRODUCTION

Guillain‐Barré syndrome (GBS) is an immune‐mediated polyradiculoneuropathy and the most common cause of acute flaccid paralysis worldwide.^1^ GBS is usually preceded by an infection, and several pathogens have been associated with GBS in case‐control studies, including *Campylobacter jejuni*, hepatitis E virus (HEV), cytomegalovirus (CMV), Epstein‐Barr virus (EBV), and *Mycoplasma pneumoniae*.^2^, ^3^, ^4^, ^5^ During the Zika virus (ZIKV) epidemic in 2015‐2016 in Latin America, an increased incidence of GBS patients was observed and an association between ZIKV and GBS has later been confirmed.^6^, ^7^, ^8^

ZIKV is a flavivirus that is transmitted by the *Aedes aegypti* mosquito. Other arthropod‐borne viruses (arboviruses) transmitted by the same mosquito, including dengue virus (DENV) and chikungunya virus (CHIKV), have also been associated with GBS, although evidence of an association is limited in comparison with ZIKV.^6^, ^9^, ^10^, ^11^, ^12^, ^13^, ^14^, ^15^, ^16^, ^17^, ^18^ Most studies on DENV and GBS are limited to case series,^10^, ^18^, ^19^, ^20^, ^21^, ^22^ although two surveillances studies^17^, ^18^ showed a temporal association between the incidence of GBS and DENV, and one case‐control study provided evidence of an association between GBS and DENV.^23^ Several studies have linked clusters of GBS cases with outbreaks of CHIKV,^15^, ^24^, ^25^, ^26^ and a case–control study^9^ demonstrated that CHIKV is a risk factor for GBS. Arboviruses have been increasingly recognized as a global health threat, as their geographic distribution has spread dramatically over the past decades.^12^, ^27^, ^28^ Roughly half of the world's population is currently living in areas at risk for transmission of these viruses, and especially countries in Latin America and Southeast Asia are at risk.^29^

Previous studies that demonstrated a link between GBS and ZIKV or other arboviruses were carried out during epidemic phases of viral transmission, and it is unknown whether these viruses also play a role in the occurrence of GBS in endemic phases. Another aspect of arbovirus‐related GBS that has not been illuminated is the possible role of coinfections with other known triggers of GBS, as most previous studies only tested for arbovirus infections. Furthermore, the underlying pathophysiology and the role of antibodies to specific gangliosides and other glycolipids on the nerve axon has not been uniformly demonstrated for GBS related to arboviruses.^26^, ^30^, ^31^, ^32^, ^33^

The International Guillain‐Barré syndrome Outcome Study (IGOS) is an international observational prospective cohort study on the disease course and outcome of GBS patients.^34^ The protocol and infrastructure of this study were used and adapted to develop a case–control study (“IGOS‐Zika study”) to investigate the association between GBS and arboviruses, and specifically whether these infections drive the occurrence of GBS beyond the peaks of epidemics. Samples were tested for a broad range of infections that are known to trigger GBS and for antibodies against glycolipids to investigate the role of coinfections and anti‐glycolipid antibodies in arbovirus‐related GBS.

## 2 METHODS

### 2.1 Study design

The study protocol of IGOS has been published elsewhere.^34^ This protocol was adapted to investigate the association between arbovirus infections and GBS. Additional questions regarding immunization history and preceding symptoms and signs of arbovirus infections were collected. Where possible, two hospital‐based controls were collected for every case. Controls were sex‐ and age‐matched (age difference <10 years) and were treated in the same hospital and collected within 10 days of the included case. Controls were excluded if they had been diagnosed with GBS 1 year prior or if they were admitted for a (post‐)infectious disorder. The same questions on arbovirus history and a serum sample were collected from the controls. Otherwise, the protocol was identical to the original IGOS protocol. Patients were enrolled in two study sites in Brazil, four sites in Argentina, and one site in Malaysia. The IGOS study (MEC‐2011‐477) and the amendment of the study protocol (NL38706.078.11) were approved by the review boards of Erasmus MC University Medical Center, Rotterdam, The Netherlands. The study protocol was also approved by the local institutional review boards of all participating hospitals or universities. Written informed consent was obtained from all patients or their legal representatives.

### 2.2 Data collection

Data were collected on demography, antecedent events, and neurological symptoms and signs of GBS at study entry and at 1, 4, and 26 weeks.^34^ Additional collection of data at weeks 2, 8, 13, and 52 was optional. Muscle strength was recorded by the Medical Research Council (MRC) score and disability by the GBS disability score.^35^, ^36^ Disease nadir was defined as the first visit that the lowest MRC sum score was found during the first 4 weeks from study entry. When there was no muscle weakness, the GBS disability score was used instead. The results of routine cerebrospinal fluid (CSF) examination and nerve conduction studies were collected. To determine the electrophysiological subtype, raw data of the first nerve conduction study, local reference values, and an algorithm were used to classify each nerve conduction study according to the criteria of Hadden et al by two independent clinical neurophysiologists (SA, JD).^37^ Patients were categorized according to the Brighton Collaboration criteria based on the available data.^38^ Insufficient data were available to categorize the Miller Fisher syndrome (MFS) patients according to the published criteria, and all patients with clinical variants of GBS without limb weakness were categorized as Level 4. The ability to walk at 6 months was used to determine the outcome. For patients with missing data at the 6‐month visit, who were able to walk independently at the previous visit (week 13 or week 8), this visit was used to determine the outcome.

### 2.3 Diagnostic virology and bacteriology

All patients and controls with available serum samples were tested for a recent infection with *C jejuni*, HEV, *M pneumoniae*, CMV, EBV, DENV, ZIKV, and CHIKV. Serum samples collected at entry or week 1 were used where possible; otherwise, samples collected at week 2 or 4 were used. Antibodies against *C jejuni* were determined using an indirect enzyme‐linked immunosorbent assay (ELISA) for IgG and antibody class capture ELISAs for IgM and IgA antibodies, as previously described.^39^ IgM and IgG antibodies against HEV and *M pneumoniae* were determined using commercially available ELISAs (Wantai, Beijing, PR China, respectively, Serion ELISA classic *M pneumoniae*, Serion GmbH, Würzburg, Germany). The presence of IgM and IgG antibodies and IgG avidity against CMV and of VCA IgM and viral capsid antigen (VCA) IgG and EBV nuclear antigen (EBNA) was determined by LIAISONXL (DiaSorin, Italy), a semi‐automated system, which uses chemiluminescent immunoassay (CLIA) technology for detection of antibodies. The presence of IgM and IgG antibodies against ZIKV and DENV were determined using commercially available ELISA (EuroImmun, Lübeck, Germany). The presence of IgM and IgG antibodies against CHIKV was determined using a commercially available ELISA (Novatec), and immunofluorescence was performed to verify the presence of IgM. Immunofluorescence was leading in the interpretation of the results. In all patients that were IgM‐ or IgG‐positive against ZIKV, a virus neutralization test (VNT) was performed to differentiate between a recent DENV and ZIKV infection.^40^ In general, IgM positivity is a good marker for a recent arbovirus infection, as studies have shown that ZIKV, CHIKV, and DENV IgM become positive starting the first week after onset of symptoms and usually persist for up to 2‐3 months.^41^, ^42^, ^43^ Evidence of a recent infection was defined as IgM positivity for *M pneumoniae* and HEV, and IgM and/or IgA positivity for *C jejuni*. For CMV, IgM positivity with negative IgG or IgG with low avidity, and for EBV, VCA IgM, and VCA IgG positivity with negative EBNA IgG was considered indicative of a recent infection. For ZIKV, IgM positivity confirmed by VNT, and for CHIKV, IgM positivity in immunofluorescence was considered indicative of a recent infection. For DENV, NS1 positivity was considered indicative of a recent (re)infection as well as the combination of IgM and IgG positivity. Low‐positive or borderline IgM with positive IgG was considered indicative of a previous infection (with possible reinfection with a different DENV strain) (Table S1).

### 2.4 Anti‐glycolipid serology

Sera were tested with ELISA for IgG and IgM antibodies against GM1, GM2, GA1, GD1a, GD1b, GT1a, GQ1b, and GD3, and using combinatorial glycoarray for IgM and IgG anti‐glycolipid antibodies against GM1, GM2, phosphatidylserine, GA1, GD1a, GD1b, GT1a, GQ1b, GD3, GalC, lactosylceramide, and sulfatide, plus their possible heterodimeric complexes.^44^, ^45^ Combinatorial glycoarray was performed using a thin‐layer chromatography autosampler, which spotted glycolipids and glycolipid combinations onto in‐house‐made glass slides containing a polyvinylidene difluoride (PVDF) membrane.^46^ Antibodies were detected using AF647‐conjugated goat anti‐human IgM and Cy3‐conjugated goat anti‐human IgG (Jackson ImmunoResearch). Fluorescent intensity was measured using the appurtenant LuxScan software. The mean and SD were calculated for each glycolipid (−complex) using the fluorescent intensities of the control patients. Fluorescent intensities were considered positive if more than the mean plus three times the SD.

### 2.5 Statistical analysis

We used SPSS Statistics 21.0 for data analysis. Continuous data are presented as medians with interquartile ranges (IQRs) and dichotomized or categorical data as numbers and proportions. We used the Mann‐Whitney *U* test and Kruskal‐Wallis test to compare continuous data and the χ^2^‐test or Fisher's exact test to compare proportions. A two‐sided *P‐value* of <0.05 was considered significant. For the case‐control analysis, crude odds ratios were calculated (not matching for pairs) using contingency tables, and 95% confidence interval were calculated according to Altman, 1991.^47^, ^48^ The Cox proportional hazards model was used for the individually paired case‐control analysis (SPSS COXREG function), adjusting for age and sex.^49^, ^50^ We used *R* version 3.6.1., packages dplyr 1.0.5 and ggplot2 3.3.2 for the development of the heatmaps. Raw data were clustered based on a distance matrix using Pearson's correlation and hierarchical cluster algorithm (Ward.2D) and clipped at a 10 000 upper limit.^51^

## 3 RESULTS

**FIGURE 1:**
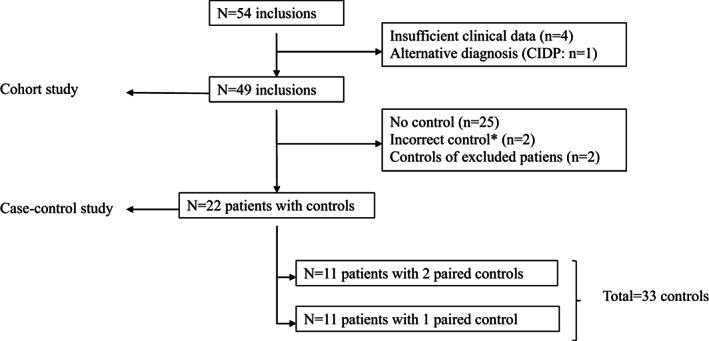
Flowchart of inclusions in cohort and case‐control part of the analysis. *Family control (brother) instead of hospital control (n = 1), hospital control admitted with Alzheimer's and chikungunya fever (n = 1)

In total, 54 patients were included between July 2017 and December 2019. Five patients were excluded, four because of insufficient clinical data and one because of an alternative diagnosis (chronic inflammatory demyelinating polyradiculoneuropathy). For 22 of the remaining 49 patients, paired controls were collected, and they were included in the case‐control analysis part of the study (Figure 1). Demographic and clinical features, ancillary investigations and outcome of the full cohort are described in Table 1.

**TABLE 1:** Demography, clinical features at entry, and outcome of the full cohort of patients with GBS

	All cases (n = 49)
Sex (male)	32 (65)
Age (years)	42 (23‐57)
<18 y old	7 (14)
Country of inclusion	
Brazil	31 (63)
Argentina	7 (14)
Malaysia	11 (22)
Antecedent event—onset weakness (days)	7 (4‐15)
Antecedent symptom (any)	36 (74)
Fever	20/36 (56)
Respiratory tract infection^a^	15/36 (42)
Gastro‐intestinal infection^b^	18/36 (50)
Rash	4/36 (11)
Cranial nerve deficits	29/48 (60)
Oculomotor	10/48 (21)
Facial	18/48 (38)
Bulbar	10/48 (21)
Limb weakness	37/48 (77)
MRC sum score	45 (32‐58)
Hypo‐/areflexia	42/48 (88)
Sensory deficits^c^	23/47 (49)
Sensory symptoms	27/41 (66)
Ataxia^c^	13/41 (32)
Onset weakness—nadir (days)	10 (5‐15)
GBS clinical variant	
Sensorimotor	19/48 (40)
Pure motor	14/48 (29)
MFS (overlap)	10/48 (20)
Other	5/48 (10)
Nerve conduction studies^d^	48/49 (98)
Demyelinating	28/48 (58)
Axonal	6/48 (13)
Equivocal	13/48 (27)
Immunomodulatory treatment	44/49 (90)
IVIg	43/49 (88)
Plasmapheresis	1/49 (2)
ICU admission	20 (41)
Mechanical ventilation	12 (25)
Able to walk unaided at 6 mo^e^	28/33 (85)

*Note*: Data are presented as n/N reported (%) or median (IQR). Clinical features presented are at study entry.

^a^ Sore throat, nasal cold and/or cough.

^b^ Diarrhea or nausea/vomiting.

^c^ If “unable to examine” coded as missing.

^d^ One patient tested negative had an inexcitable EMG.

^e^ Patients able to walk at 8 or 13 wk and missing data at week 26 were included in this category.

### 3.1 Viral and bacterial serology

Evidence of a recent infection was found in 27/49 (55%) of patients and included arbovirus infections in four patients (8%), including CHIKV in two (4%), DENV and CMV in one (2%), and DENV and *C jejuni* in one patient (2%). In addition, in one patient, a low‐positive IgM, positive IgG, and negative NS1 indicated a possible reinfection with a different DENV strain, and in one patient, a borderline‐positive IgM, and positive IgG and VNT for ZIKV indicated a possible recent ZIKV infection. For the purpose of this study, these patients were not considered positive for a recent infection with these viruses. Details of serological test results for arbovirus infection‐positive cases are shown in Table S2. The patients with a recent CHIKV infection and the patient with a recent DENV and *C jejuni* infection were included in Northeast Brazil between May and July 2019. The patient with a DENV and CMV infection was included in Malaysia in August 2019 (Table 2). *C jejuni* was the most common preceding infection in 15 patients (31%), followed by *M pneumoniae* in five (10%), and one additional patient had evidence of a recent infection with both these pathogens. Evidence of a recent EBV infection was found in one patient (2%), and none of the patients had evidence of a recent HEV infection. Samples were collected at a median of 11 days (IQR 7‐19) after the onset of weakness.

**TABLE 2:** Demographic and clinical features of GBS patients with evidence of a recent arbovirus infection

	Sex, age, country	Antecedent event	Clinical features (entry)	GBS clinical variant	EMG subtype	Treatment, ICU, and ventilation	Disease nadir	Outcome last follow‐up
CHIKV^a^	male, 72 y/o, Brazil	Nasal cold (20 d prior)	Brighton Level 1. Bulbar and oculomotor palsy, limb weakness, sensory deficits, blood pressure dysfunction	MFS‐GBS overlap	Demyelinating	IVIg (5 d), admitted to ICU (7 d) and MV (3 d)	MRC‐SS =32, GBS‐DS = 5. Onset‐nadir 9 d	MRC‐SS = 60, GBS‐ DS w8 = 0 (w8 last follow‐up)
CHIKV^b^	female, 37 y/o, Brazil	Fever, joint pain, rash (4 d prior)	Brighton Level 1. Bulbar and facial palsy, limb weakness, sensory deficits, blood pressure dysfunction	Sensorimotor	Demyelinating	IVIg (5d), admitted to ICU (21 d) and MV (17 d)	MRC‐SS = 28, GBS‐DS = 5 Onset‐nadir 11 d	MRC‐SS = 58, GBS‐ DS = 4 (w8 last follow‐up)
DENV and CMV	male, 30 y/o, Malaysia	Fever, myalgia, arthralgia, headache, retro‐ocular pain (13 d prior)	Brighton Level 4. Facial palsy, sensory deficits, ataxia	Ataxic form	Demyelinating	IVIg (5 d), no ICU or MV	MRC‐SS = 60, GBS‐DS = 3 Onset‐nadir 7 d	MRC‐SS = 60, GBS‐ DS = 0 (w26 last follow‐up)
DENV and *Campylobacter jejuni*	male, 19 y/o, Brazil	Fever, diarrhea (5 d prior)	Brighton Level 2. Limb weakness	Pure motor	Axonal	IVIg (5 d), no ICU or MV	MRC‐SS = 40, GBS‐DS = 3 Onset‐nadir 5 d	MRC‐SS = 54, GBS‐ DS = 2 (w26 last follow‐up)

Abbreviations: *C. jejuni*, *Campylobacter jejuni*; CHIKV, chikungunya virus; CMV, cytomegalovirus; DENV, dengue virus; GBS‐DS, GBS disability score; ICU, intensive care unit; IVIg, intravenous immunoglobulins; MFS, Miller Fisher syndrome; MRC‐SS, MRC sum score; MV, mechanical ventilation; y/o, years old.

^a^ P40 in Figure 2.

^b^ P39 in Figure 2.

### 3.2 Clinical features, ancillary investigations, and outcome of the full cohort

The median time between onset of neurological symptoms and hospital admission was 6 days (IQR 3‐10). Lumbar puncture was done in 46/48 reported patients (96%). In 73%, an increased protein level (>0.45 g/L) and a cell count below 50 cells/μL was found (albuminocytological dissociation).^34^, ^52^ The median cell count was 1.0 (1.0‐3.5), and none of the patients had a cell count above 50 cells/μL. Nerve conduction studies were performed in 48 (98%) patients. To exclude differential diagnoses, MRI of the spinal cord was performed in eight patients and was normal in six and showed enhancement of the cauda equina in two. According to the Brighton Collaboration Criteria, 25 (51%) had Level 1, 7 (14%) Level 2, and 17 (35%) Level 4. Patients were categorized as Brighton Level 4 because of: time to nadir >28 days (n = 2), normal (n = 4) or increased tendon reflexes (n = 1), clinical variant of GBS without limb weakness (n = 9), and missing data on time to nadir (n = 1). Four of five patients with normal or increased tendon reflexes had evidence of a recent *C jejuni* infection. Nerve conduction studies showed signs typical of a poly(radiculo)neuropathy in 16/17 patients (96%), and 9/17 (53%) had an albuminocytological dissociation in the CSF.

At disease nadir, 79% of patients were unable to walk unaided (GBS disability score ≥ 3), and the median MRC sum score was 43 (IQR 31‐46). When including the patients with missing data at 6 months, but who were able to walk at week 8 or week 13 after study inclusion, 28/33 (85%) were able to walk unaided at 6 months. Eighteen of 19 patients (95%) who were followed up to 1 year or more were able to walk unaided at 1 year. One patient died due to complications of pulmonary tuberculosis 5 months after the onset of GBS.

### 3.3 Comparison of infection groups

Preceding symptoms of an infection were reported in 36 (74%) of the patients and included fever, and gastro‐intestinal and respiratory tract infection. Of the patients with preceding symptoms of an infection, 16 (44%) had no serological evidence of a recent infection. In contrast, of the 27 patients with serological evidence of a recent infection, 7 (26%) did not have preceding infectious symptoms. Antecedent events other than infectious symptoms included vaccination (n = 4) and surgery (n = 1). The types of vaccination were influenza, polio, and tetanus. All patients that reported a recent vaccination had serological evidence of a recent infection, with *C jejuni* (n = 1), *M pneumoniae* (n = 1), EBV (n = 1), and CHIKV (n = 1). The patient with surgery also had preceding infectious symptoms, including fever, gastro‐intestinal complaints, and joint pain. She was negative for the tested infections.

The clinical features of the patients with evidence of a recent arbovirus infection are shown in Table 2. The two patients with a recent CHIKV infection had different clinical variants (MFS‐overlap and sensorimotor), the same electrophysiological subtype (demyelinating), and a similar clinical progression; both were admitted to ICU and ventilated, had a low MRC sum score at nadir, but near‐complete recovery of strength at 8‐week follow‐up. One of these patients had typical antecedent symptoms of CHIKV infection, including fever, joint pain, and rash; the other reported a nasal cold 20 days prior. The patient with a recent DENV and CMV infection reported preceding symptoms of fever, myalgia, arthralgia, headache, and retro‐ocular pain and had an ataxic variant and demyelinating subtype of GBS. The patient with a recent DENV and *C jejuni* infection had preceding symptoms of a gastro‐enteritis and a pure motor variant and axonal subtype of GBS.

In patients with a recent *C jejuni* infection, gastro‐enteritis was the most common reported antecedent event (78%). The pure motor variant of GBS was most frequently reported (12/15, 80%), cranial nerve involvement was infrequent (5/15, 33%), and the MRC sum score at entry was relatively low (41 [IQR 30‐46]). Nine of 12 reported patients (75%) were able to walk unaided at 6 months. The five patients with a *M pneumoniae* infection were frequently <18 years old (2/5, 40%), and had a relatively long time between antecedent event and onset of weakness (18 days [IQR 11‐21]), and a high MRC sum score at entry (59 [IQR 56‐60]), and 2/2 reported patients had fully recovered at 8 weeks. The patient with a recent EBV infection was 9 years old, and had preceding symptoms of headache and nausea, a sensorimotor demyelinating variant, and full recovery of disability at 13‐week follow‐up. Details on the clinical features per infection group are displayed in Table S3.

### 3.4 Anti‐ganglioside antibodies

The presence of serum anti‐ganglioside antibodies (IgM and IgG) against 12 commonly studied glycolipids in GBS was tested in ELISA and combinatorial glycoarray.

In ELISA, 21 patients (43%) were positive for one or more of these antibodies (IgM or IgG), vs none of the 32 tested controls (Table 3). In patients with a CHIKV or EBV infection, no anti‐ganglioside antibodies were found in ELISA. In patients with a *C jejuni* infection, antibodies against GM1, GM2, and GD1a were most frequently reported. In the patient with a *C jejuni* and DENV infection, IgM antibodies against GM1, GM2, and IgM and IgG against GD1a were found, and in the patient with a CMV and DENV infection, IgM antibodies against GM2 were found. The presence of anti‐ganglioside antibodies (IgM or IgG) was found in patients with an axonal (4/6, 67%) as well as in patients with a demyelinating electrophysiological subtype of GBS (14/28, 50%).

**TABLE 3:** Anti‐ganglioside antibodies in serum (ELISA)

	Controls (n = 32)^a^		All cases (n = 49)		*Campylobacter jejuni* (n=15)		*Mycoplasma pneumoniae* (n = 5)	
	IgM	IgG	IgM	IgG	IgM	IgG	IgM	IgG
Any	0 (0)	0 (0)	11 (22)	15 (31)	6 (40)	9 (60)	1 (20)	1 (20)
GM1	0 (0)	0 (0)	6 (12)	5 (10)	4 (27)	4 (27)	1 (20)	0 (0)
GM2	0 (0)	0 (0)	6 (12)	1 (2)	4 (27)	1 (7)	0 (0)	0 (0)
GD1a	0 (0)	0 (0)	4 (8)	5 (10)	4 (27)	4 (27)	0 (0)	0 (0)
GD1b	0 (0)	0 (0)	1 (2)	6 (12)	0 (0)	3 (20)	0 (0)	1 (20)
GD3	0 (0)	0 (0)	0 (0)	1 (2)	0 (0)	0 (0)	0 (0)	0 (0)
GQ1b	0 (0)	0 (0)	0 (0)	3 (6)	0 (0)	0 (0)	0 (0)	0 (0)

^a^ In 32/35 controls, sufficient serum sample was available for anti‐ganglioside antibody testing.

**FIGURE 2:**
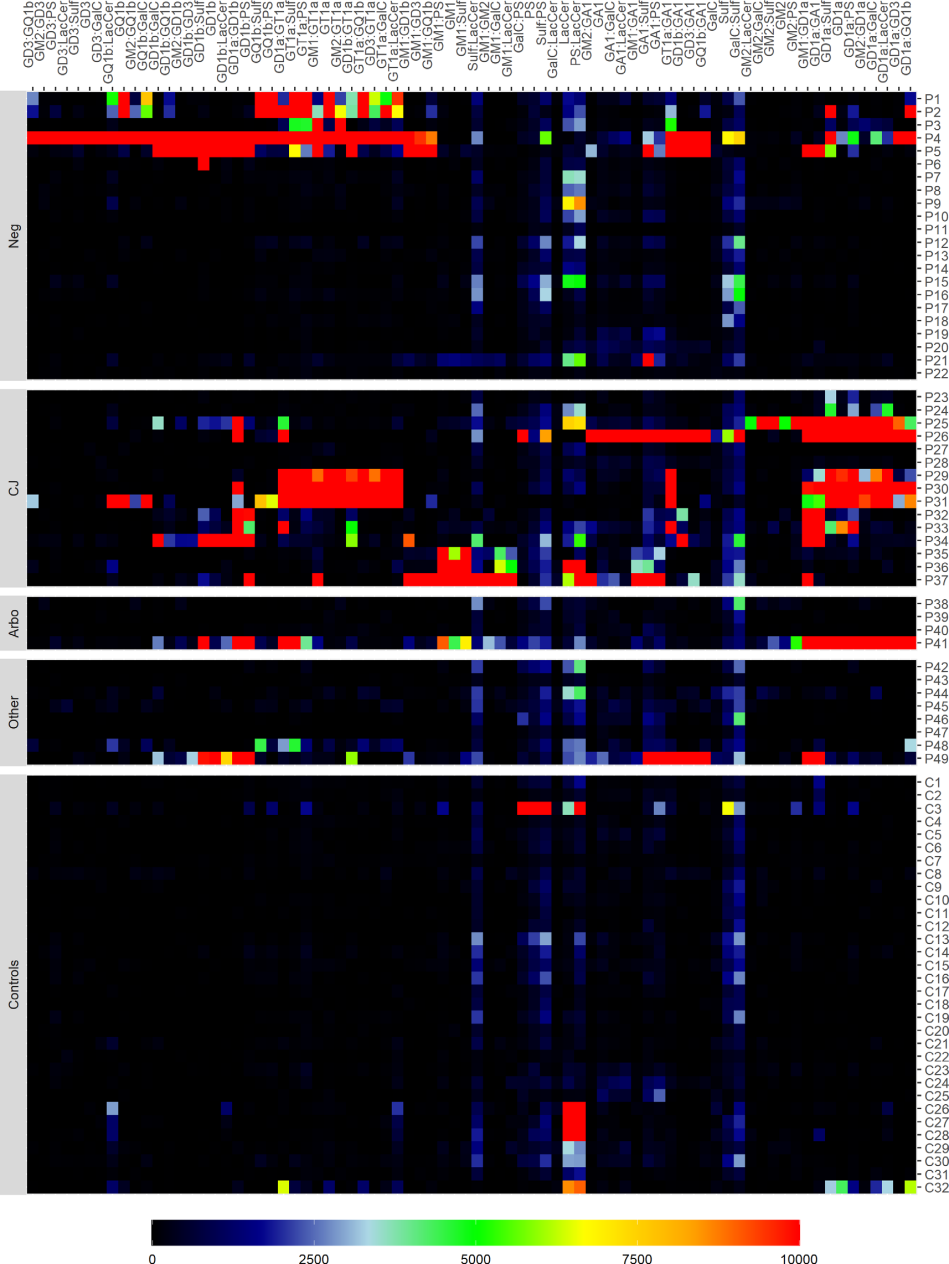
Heatmap of IgG antibody binding to glycolipids as assessed by glycoarray. Each row presents one patient (P1‐P49) or control (C1‐C23); each column presents one of the tested glycolipid antibodies (single or in complex). Raw data were was clustered based on a distance matrix using Pearson's correlation and hierarchical cluster algorithm, and clipped at a 10 000 upper limit

In glycoarray, 19 patients (39%) were positive for IgM and 25 (51%) for IgG antibodies against single glycolipids, and 26 patients (53%) were positive for IgM and 36 (74%) for IgG antibodies against glycolipids in complexes. In contrast, of the 32 controls, 2 (6%) were positive IgM and 6 (19%) for IgG antibodies against single glycolipids, and 11 (34%) for IgM and 10 (31%) for IgG antibodies against glycolipids in complexes. In Figure 2, glycoarray findings are visualized in a heatmap. Binding of IgG antibodies to glycolipids is clearly lower in cases vs controls although some reactivity against GalC, lactosylceramide, and sulfatide is seen in both cases and controls. Similar to the ELISA results, no or only low reactivity was found in patients with (arbo)virus infections. The patient with a recent CHIKV infection that had a MFS‐GBS overlap variant (P40) was positive for IgG and IgM antibody binding to GD3 in complex with several other glycolipids, including GQ1b, but binding was low and not visible in the heatmap (Figure 2). In the other patients with a recent CHIKV infection (P39), no antibody binding to glycolipids was found. In one patient with *M pneumoniae* infection and a sensorimotor variant of GBS (P49), reactivity was found against complexes with GD1a, GD1b, GD3, and GQ1b. In patients with a *C jejuni* infection, a large variety of reactivities were found, but clusters were mostly seen in complexes with GM1, GD1a, and GD1b. The patient with a *C jejuni* and DENV infection (P41) showed complex reactivity similar to that of other patients with a *C jejuni* infection.

### 3.5 Case‐control study

In total, 35 paired controls were collected of 23 cases. One of these cases was excluded because of an alternative diagnosis, leaving 22 patients with 33 paired controls for the paired case‐control analysis (Table S4). None of the cases or controls included in this analysis had evidence of a recent infection with ZIKV, CHIKV, or EBV. Calculated crude odds ratio and adjusted odds ratio of recent infections were not significant.

We also performed an unpaired case‐control analysis, comparing all 49 cases to all 35 controls (Table 4). Although all infections occurred more frequently in cases vs controls, calculated crude odds ratio were not significant. Evidence of a recent infection with DENV, CHIKV, CMV, or EBV was only found in cases. Furthermore, two cases had a possible recent arbovirus infection (one ZIKV infection and one DENV reinfection), and in none of the controls were such borderline results found.

**TABLE 4:** Unpaired case‐control analysis

Evidence of recent infection^a^	Controls (n = 35)	Cases (n = 49)	Crude odds ratio (CI)^b^	*P*‐value
Dengue virus	0/35 (0%)	2 (4%)	3.737 (0.174‐80.290)	0.3996
Chikungunya virus	0/31 (0%)	2 (4%)	3.316 (0.154‐71.403)	0.4440
*Campylobacter jejuni*	6/30 (20%)	18 (37%)	2.323 (0.799‐6.748)	0.1215
*Mycoplasma pneumoniae*	4/31 (13%)	7 (14%)	1.125 (0.301‐4.212)	0.8612
Cytomegalovirus	0/27 (0%)	1/46 (2%)	1.813 (0.0713‐46.089)	0.7185
Epstein‐Barr virus	0/27 (0%)	1/46 (2%)	1.813 (0.0713‐46.089)	0.7185

*Note*: Proportions are shown as number positive/number tested.

^a^ Zika virus and hepatitis E virus are not displayed in this table as none of the cases and none of the controls had evidence of a recent infection with these viruses. Not all cases and controls were tested for all infections.

^b^ Odds ratio was calculated using the Haldane‐Anscombe correction if one of the two groups had zero subjects.

## 4 DISCUSSION

Previous studies conducted during epidemic phases of arboviral transmission have demonstrated evidence of an association between GBS and ZIKV, CHIKV, and DENV. However, literature on the occurrence of arbovirus infections in GBS patients during endemic phases of transmission is limited.^53^ In this observational multinational cohort and case‐control study on GBS in relation to arbovirus infections, we found that these infections do occur at low rates in GBS patients during endemic phases of viral transmission. Of the 49 patients included in the study, a recent arbovirus infection was found in four cases (8%) that were collected in Northeast Brazil and Malaysia during times when no epidemics of arbovirus infections were reported, and included CHIKV (n = 2) and DENV (n = 2). Two additional patients had evidence of a possible recent infection with ZIKV and DENV. In contrast, we did not find evidence of a (possible) recent arbovirus infection in any of the 35 controls. Odds ratio did not significantly differ between cases and controls, most likely because our study was underpowered, indicated by the broad confidence intervals. The absence of ZIKV‐related GBS in the current study, conducted in a period of low viral transmission, is in accordance with the results of a meta‐analysis that estimated the overall risk of reported GBS at 2.0 (95% CI 0.5‐4.5) per 10 000 ZIKV cases.^54^ Nevertheless, this estimated risk is magnitudes higher than the annual global incidence of GBS (±1‐2 cases per 100 000 person‐years), indicating the potential of ZIKV to cause large outbreaks of GBS during epidemics. The risk of GBS after CHIKV or DENV has not been defined in detail, but based on our results, it is likely that these infections may also be an infrequent trigger of GBS during endemic phases of transmission. No data on IgM seroprevalence are available during the time period (2017‐2019) and in the specific regions of our study. A seroprevalence study performed in a different area in Brazil in 2018 showed an IgM seroprevalence of 5% for CHIKV and 2% for DENV and ZIKV.^55^ One study from Malaysia performed between 2012 and 2017 showed 0.6%‐2.2% seropositivity for ZIKV neutralizing antibodies,^56^ and another study performed in 2015 in a rural area showed ±11% IgM seroprevalence of DENV.^57^ We were not able to find reliable data on CHIKV IgM seroprevalence in Malaysia or of any of the three arboviruses for Argentina. Although the proportion of positive cases found in this study is higher than most of these seroprevalence studies, we are unable to draw any conclusions due to the differences in the study population.

We also tested our cohort for other infections that have previously been associated with GBS and found evidence of a recent *C jejuni* infection in 18 (37%), *M pneumoniae* in 7 (14%), CMV in one (2%), and EBV in one (2%). Infections with *C jejuni* were specifically frequent in Brazilian patients in our study. Studies on GBS in Brazil outside of the ZIKV pandemic are scarce, and other infections have rarely been tested.^58^, ^59^ These results indicate that, as in other countries, *C jejuni* is the most common trigger in Brazil. The two patients with a recent DENV infection also had evidence of another infection: one with *C jejuni* and one with CMV. It is not clear what the significance of these coinfections is. The presence of several recent infections may cause a more severe immune response that increases the risk of development of GBS,^60^ or polyclonal B‐cell activation as a response to one infection may lead to false‐positive serologic test results for other pathogens.^61^, ^62^ Nevertheless, this finding indicates that previous studies only testing for arboviruses may have missed patients who also had evidence of another infection associated with GBS.

In our study, preceding symptoms of an infection were only partly correlated with the serological evidence of a recent infection. In almost half of the patients who had preceding symptoms of an infection, no serological evidence of a recent infection was found. It may be that part of these cases were false‐negative for the tested infections, as we were not able to perform PCR or culture and therefore may have missed some cases that did not (yet) mount a detectable serological response. Alternatively or in addition, some of these patients may have had infections that were not tested for in this cohort, which may include *Haemophilus influenzae* or varicella zoster virus, as these have been linked to GBS in some previous studies.^63^, ^64^, ^65^ However, it is important to note that in 26% of the patients *with* serological evidence of an infection, no preceding infectious symptoms were reported, which may indicate minor symptoms or asymptomatic infection. Furthermore, in all patients who reported a vaccination, serological evidence of an infection was found. This is expected, as for most vaccines no evidence exists of an association with GBS, and it highlights the importance of also investigating other infectious causes in patients developing GBS in the weeks after receiving a vaccine.^66^, ^67^

The clinical and electrophysiological profile of GBS in relation to the preceding infections confirmed findings from previous studies. The patients with a preceding *C jejuni* infection frequently had a pure motor variant and axonal electrophysiological subtype and more severe muscle weakness and slower recovery.^5^, ^68^ A minority of these patients had normal or increased tendon reflexes, as has been reported in other patients with *C jejuni‐*associated GBS.^69^ Patients with a recent *M pneumoniae* infection were younger, and patients with preceding virus infections, including those with recent CHIKV infection, generally had a demyelinating electrophysiological subtype of GBS and a relatively fast recovery.^2^, ^5^, ^26^, ^70^ Both patients with CHIKV infection were admitted to the ICU and ventilated. In previous studies on arbovirus‐related GBS, higher proportions of ICU admission and mechanical ventilation were found compared to other GBS cohorts.^23^, ^26^, ^71^ This may indicate that arbovirus‐related GBS is associated with a more severe initial disease course and/or respiratory insufficiency, but patient numbers are too small to draw conclusions.

Serology of anti‐ganglioside antibodies clearly showed higher reactivities in patients compared to controls, both in ELISA and in glycoarray, confirming the role of these antibodies in the pathophysiology of GBS.^2^, ^30^ The patients with a recent *C jejuni* infection mainly displayed binding of GM1, GM2, GD1a, and GT1a, as has been reported previously.^72^, ^73^, ^74^ In one of the patients with a recent CHIKV infection, low binding of GD3 antibodies in complex was found, and in the other CHIKV‐positive case, no binding was found. The patient with a recent DENV and *C jejuni* infection had an anti‐glycolipid complex reactivity similar to that of the patients with a *C jejuni* mono‐infection, and in the patient with a recent DENV and CMV infection, IgM antibodies against GM2 were found, similar to previously published cases of CMV‐related GBS.^75^, ^76^ This is in line with a previous study from Northeast Brazil where we did not find a specific anti‐ganglioside antibody profile related to arbovirus infections.^26^ Although a study on ZIKV‐related GBS in French‐Polynesia demonstrated antibody activity against GD1a, this has not been replicated in any study on GBS conducted during the Latin American ZIKV epidemic.^31^ In general, anti‐ganglioside antibodies have rarely been demonstrated in GBS patients with preceding virus infections, indicating that the underlying pathophysiology may be different from bacterium‐related GBS.

The fact that the anti‐ganglioside antibody profile of the patients with a DENV was more typical for their coinfection suggests that the CMV or *C jejuni* infection were the actual trigger of GBS in these cases, and the DENV infection was a coincidental finding. The patient with a *C jejuni* and DENV infection also had a clinical profile most compatible with a *C jejuni* infection, with preceding diarrhea and a pure motor axonal variant of GBS. This is similar to findings of a study from Bangladesh conducted during an endemic phase of ZIKV transmission, where 9/18 ZIKV‐positive GBS cases also had evidence of a recent *C jejuni* infection, and a clinical phenotype typical for that infection.^77^

Our study has several limitations. Most importantly, the case‐control study was underpowered. It was not always possible in clinical practice to collect two paired controls for every case, as per the original protocol, an unfortunate but unavoidable feat in a multinational study. Second, participating centers were mostly academic or teaching hospitals, and inclusion of patients may have been biased towards more complicated or severe cases. Third, although we used sophisticated serological testing to identify the presence of arbovirus and other preceding infections, we were not able to perform PCR (for the viruses) or culture (for the bacteria) due to sample and cost limitations, and may have missed patients that did not (yet) mount a serological response.

In conclusion, we found that preceding infections with CHIKV and DENV occur in GBS patients outside of epidemics, although not significantly more often than in controls. Broad serological testing and anti‐ganglioside antibody diagnostics, as well as clinical and electrophysiological findings, may be helpful in determining the actual trigger in GBS patients with coinfections. Larger studies on arbovirus‐related GBS are necessary to further study the association with GBS in endemic phases of transmission.

## CONFLICT OF INTEREST

SEL, CYT, AAvdE, RR, SCF, SA, MRB, SMBJ, JD, LdK, LLC, CM, WMJr, DFC, AS, BT, and MED report no conflicts of interest. RH reports grants from GBS‐CIDP Foundation International and Health~Holland and is editorial board member of the *Journal of the Peripheral Nervous System*. BCJ received grants from Grifols, CSL‐Behring, Annexon, Prinses Beatrix Spierfonds, Hansa Biomedical, and GBS‐CIDP Foundation International and is on the Global Medical Advisory Board of the GBS CIDP Foundation International.

## Supporting information


**Appendix S1.** Supporting Information.
