# Supplementary material for: Antecedent infections in Guillain‐Barré syndrome in endemic areas of arbovirus transmission: A multinational case‐control study
Source: J Peripher Nerv Syst. 2021 Sep 30;26(4):449–60. doi: 10.1111/jns.12469 (PMC9291970; doi:10.1111/jns.12469)
Supplement: Supplementary file 1 — Appendix S1. Supporting Information. [file JNS-26-449-s001.docx]

**SUPPLEMENTARY MATERIAL**

**Methods**

*Diagnostic virology and bacteriology*

All virological and *M. pneumoniae* diagnostic testing was done at the Viroscience Department of the Erasmus University Medical Center, Rotterdam, The Netherlands. *C. jejuni* diagnostic testing was done at the Reinier de Graaf Gasthuis, Delft, The Netherlands. In three patients and three controls insufficient sample was available for EBV and CMV tests. Of an additional six controls collected in Argentina, no serum samples were transported to The Netherlands for serological testing. Six of these controls were tested locally for several preceding infections and these results were included for the case-control analysis.

The presence of anti-*C. jejuni* antibodies was expressed as a ratio of optical density between a test sample and a reference serum sample which was included in all tests

**Supplementary Table 1. Definition or recent infection**

|  | **Recent infection** |
| --- | --- |
| Zika virus | IgM+ AND VNT + |
| Dengue virus | NS1+ *OR*  IgM+ AND IgG+ |
| Chikungunya virus | IgM+ |
| Cytomegalovirus | IgM+ AND IgG- OR IgG+ with low avidity |
| Epstein-Barr virus | VCA IgM_ and VCA IgG+ with EBNA- |
| Hepatitis E virus | IgM+ |
| *Campylobacter jejuni* | IgM+ OR IgA+ |
| *Mycoplasma pneumoniae* | IgM+ |

VNT= virus neutralization test ,VCA= viral capsid antigen, EBNA= EBV nuclear antigen

*Anti-glycolipid serology*

Combinatorial glycoarray analysis and ELISA of serum samples was performed at the Immunology Department of the Erasmus University Medical Center, Rotterdam, The Netherlands University, to detect IgM and IgG antibodies against glycolipids. Combinatorial glycoarray was done using a thin-layer chromatography autosampler (ATS4, Camag) and the winCATS software glycolipids and glycolipid-combinations were spotted onto in-house made glass slides (VWR, Radnor, Pennsylvania, USA) containing a polyvinylidene difluoride (PVDF) membrane (Immobilon® FL membrane, 0.45 μm, Merck Millipore Ltd., Watford, UK). Membranes were blocked with 2% BSA PBS and subsequently incubated with the GBS patient or control patient sera (1:100) or plasma (1:100). After washing, slides were incubated with goat α-human IgG-Cy3 (1:1000) (Jackson ImmunoResearch, West Grove, Pennsylvania, USA) and goat α-human IgM-AF647 (1:1000) (Jackson ImmunoResearch). Washed and dried (O/N) slides were scanned using the LuxScan™ 10K Microarray Scanner (CapitalBioTech, Beijing, China). All settings were pre-defined and optimized on forehand. The mean fluorescent signal of the 1:9 chloroform:methanol was subtracted of all fluorescent signals.

**Supplementary Table 2. Details of serological test results for arbovirus infection positive cases**

| **Patient #** | **Time sample** | **Diagnosis** | **DENV** | | | | | | **ZIKV** | | | | | **CHIKV** | | | |
| --- | --- | --- | --- | --- | --- | --- | --- | --- | --- | --- | --- | --- | --- | --- | --- | --- | --- |
|  |  |  | **IgM** | **Ratio** | **IgG** | **Ratio** | **NS1** | **Ratio** | **IgM** | **Ratio** | **IgG** | **Ratio** | **VNT** | **IgM** | **Ratio** | **IgG** | **Ratio** |
| P38 | 6 days | Recent DENV | pos | 4.24 | pos | 3.08 | neg | -0.01 | pos | 1.55 | pos | 1.87 | neg | neg | 0.74 | pos | 0.88 |
| P39 | 11 days | Recent CHIKV | neg | 0.62 | pos | 3.40 | neg | 0.00 | neg | 0.13 | pos | 1.96 | N/A | pos | 3.94 | pos | 4.63 |
| P40 | 9 days | Recent CHIKV, past DENV | pos | 1.70 | pos | 3.24 | neg | 0.00 | neg | 0.10 | neg | 0.48 | N/A | pos | 3.82 | pos | 4.08 |
| P41 | 5 days | Recent DENV | neg | 0.36 | pos | 2.15 | pos | 1.93 | neg | 0.10 | neg | 0.38 | N/A | neg | 0.41 | neg | 0.43 |

Time sample= time between onset GBS and sample collection

**Supplementary Table 3.**

|  | ***C. jejuni* (n=15)** | ***M. pneumoniae* (n=5)** | ***C. jejuni* & *M. pneumoniae* (n=2)** | **EBV (n=1)** | **Negative (n=22)** |
| --- | --- | --- | --- | --- | --- |
| Sex (male) | 10 (67) | 4 (80) | 2 (100) | 1 (100) | 12 (55) |
| Age (years) | 37 (24-50) | 33 (17-37) | 9 and 20 | 9 | 56 (37-60) |
| <18 years old | 2 (13) | 2 (40) | 1 (50) | 1 (100) | 1 (5) |
| Country of inclusion |  |  |  |  |  |
| Brazil | 10 (67) | 4 (80) | 2 (100) | 1 (100) | 11 (50) |
| Argentina | 3 (20) | 1 (20) | 0 (0) | 0 (0) | 3 (14) |
| Malaysia | 2 (13) | 0 (0) | 0 (0) | 0 (0) | 8 (36) |
| Antecedent event - onset weakness (days) | 7 (4-9) | 18 (11-21) | 15 and 17 | 5 | 7 (4-13) |
| Antecedent symptom (any) | 9 (60) | 4 (80) | 2 (100) | 1 (100) | 16 (73) |
| Fever | 6/9 (67) | 1/4 (25) | 1 (50) | 0 (0) | 9/16 (56) |
| Respiratory tract infection^a^ | 1/9 (11) | 3/4 (75) | 2 (100) | 0 (0) | 8/16 (50) |
| Gastro-intestinal infection^b^ | 7 (78) | 1/4 (25) | 0 (0) | 1 (100) | 8/16 (50) |
| Rash | 1/9 (11) | 1/4 (25) | 0 (0) | 0 (0) | 1/16 (6) |
| Cranial nerve deficits | 5 (33) | 1 (20) |  |  | 17 (77) |
| Oculomotor | 0 (0) | 0 (0) | 1 (50) | 0 (0) | 9/21 (38) |
| Facial | 3 (20) | 1 (20) | 1 (50) | 1 (100) | 10/21 (48) |
| Bulbar | 3 (20) | 0 (0) | 0 (0) | 0 (0) | 5/21 (24) |
| MRC sum score | 41 (30-46) | 59 (56-60) | 56 and 60 | 38 | 50 (22-60) |
| Sensory deficits^c^ | 2 (13) | 3 (60) | 2 (100) | 1 (100) | 13/21 (62) |
| Ataxia^c^ | 0 (0) | 3 (60) | 1 (50) | 0 (0) | 7/17 (41) |
| Autonomic dysfunction^d^ | 5 (33) | 3 (60) | 1 (50) | 1 (100) | 12 (55) |
| Onset weakness- nadir (days) | 9 (5-13) | 11 (11-13) | 9 and 19 | 10 | 10 (6-17) |
| GBS variant |  |  |  |  |  |
| Sensorimotor | 3 (20) | 2 (40) | 1 (50) | 1 (100) | 11/21 (52) |
| Pure motor | 12 (80) | 0 (0) | 0 (0) | 0 (0) | 1/21 (5) |
| MFS (overlap) | 0 (0) | 1 (20) | 1 (50) | 0 (0) | 7/21 (33) |
| Other | 0 (0) | 2 (40) | 0 (0) | 0 (0) | 2 (10) |
| Nerve conduction studies^e^ |  |  |  |  |  |
| Demyelinating | 8 (57) | 1 (20) | 1 (50) | 1 (100) | 14 (64) |
| Axonal | 3 (21) | 1 (20) | 0 (0) | 0 (0) | 1 (5) |
| Equivocal | 3 (21) | 3 (60) | 1 (50) | 0 (0) | 6 (27) |
| Treatment |  |  |  |  |  |
| IVIg | 14 (93) | 5 (100) | 2 (100) | 1 (100) | 18 (82) |
| Plasmapheresis | 0 (0) | 0 (0) | 0 (0) | 0 (0) | 1 (5) |
| ICU admission | 7 (47) | 1 (20) | 0 (0) | 1 (100) | 10 (46) |
| Mechanical ventilation | 2 (13) | 0 (0) | 0 (0) | 0 (0) | 8 (36) |
| Able to walk unaided at 6 months^f^ | 9/12 (75) | 2/2 (100) | 2 (100) | 1 (100) | 11/13 (85) |

Data are presented as n/N reported (%) or median (IQR). Clinical features presented are at study entry. ^a^Sore throat, nasal cold and/or cough, ^b^Diarrhea or Nausea/vomiting, ^c^If ‘unable to examine’coded as missing, ^d^Including: blood pressure dysfunction (n=20), gastro-enteric dysfunction (n=7), and cardiac arrhythmias (n=5).  ^e^One patient tested negative had an inexcitable EMG, ^f^Patients able to walk at 8 or 13 weeks and missing data at week 26 were included in this category

**Supplementary Table 4**

| **Viral and bacterial serology: paired case-control analysis** | | | | |
| --- | --- | --- | --- | --- |
| **Evidence of recent infection^a^** | **Cases**  **(n=22)** | **Controls**  **(n=33)** | **Crude odds ratio (CI)** | **Adjusted odds ratio^b^ (CI)** |
| Dengue virus | 1 (5%) | 0 (0%) | 169.48 (0.00-1.44E+10) | 51625.72 (0.00-2.89E+99) |
| *C. jejuni* | 7 (32%) | 6/28 (21%) | 1.53 (0.42-5.57) | 2.08 (0.49-8.79) |
| *M. pneumoniae* | 1 (5%) | 4/29 (14%) | 0.18 (0.00-226.34) | 0.00 (0.00 - 9.22E+44) |
| Cytomegalovirus | 1/21 (5%) | 0/28 (0%) | 169.48 (0.00-1.44E+10) | 45568.80 (0.00-6.77E99) |

^a^Zika virus, chikungunya virus, Hepatitis E virus and Epstein-Barr virus are not displayed in this table as none of the cases and none of the controls in the paired case-control analysis had evidence of a recent infection with these viruses.

^b^Controlled for age and sex
